# Dynamical Coordination of Hand Intrinsic Muscles for Precision Grip in Diabetes Mellitus

**DOI:** 10.1038/s41598-018-22588-z

**Published:** 2018-03-12

**Authors:** Ke Li, Na Wei, Mei Cheng, Xingguo Hou, Jun Song

**Affiliations:** 1 Laboratory of Motor Control and Rehabilitation, Institute of Biomedical Engineering, School of Control Science and Engineering, Shandong University, 17923 Jingshi Aveue, Jinan, 250061 Shandong China; 2 Department of Geriatrics, Qilu Hospital, Shandong University, No. 107, Wenhuaxi Road, Jinan, 250012 Shandong China; 3 Suzhou Institute of Shandong University, No.388 Ruoshui Road, Suzhou, 215123 Jiangsu China; 4 Department of Endocrinology, Qilu Hospital, Shandong University, No. 107, Wenhuaxi Road, Jinan, 250012 Shandong China

## Abstract

This study investigated the effects of diabetes mellitus (DM) on dynamical coordination of hand intrinsic muscles during precision grip. Precision grip was tested using a custom designed apparatus with stable and unstable loads, during which the surface electromyographic (sEMG) signals of the abductor pollicis brevis (APB) and first dorsal interosseous (FDI) were recorded simultaneously. Recurrence quantification analysis (RQA) was applied to quantify the dynamical structure of sEMG signals of the APB and FDI; and cross recurrence quantification analysis (CRQA) was used to assess the intermuscular coupling between the two intrinsic muscles. This study revealed that the DM altered the dynamical structure of muscle activation for the FDI and the dynamical intermuscular coordination between the APB and FDI during precision grip. A reinforced feedforward mechanism that compensates the loss of sensory feedbacks in DM may be responsible for the stronger intermuscular coupling between the APB and FDI muscles. Sensory deficits in DM remarkably decreased the capacity of online motor adjustment based on sensory feedback, rendering a lower adaptability to the uncertainty of environment. This study shed light on inherent dynamical properties underlying the intrinsic muscle activation and intermuscular coordination for precision grip and the effects of DM on hand sensorimotor function.

## Introduction

Diabetes mellitus (DM) is a metabolic disorder characterized by chronic high blood glucose levels. A primary complication of DM is diabetic peripheral neuropathy (DPN), which has a prevalence from 30% to 50% of the patients with DM^1^. Conventional standpoint suggested that the symptoms of DPN mostly manifest on the lower limbs^2,3^, while a growing number of studies highlighted the effects of DPN on the upper-limbs^1,4^. Approximately 58% to 82% and 37% to 69% of patients with DM suffer from subclinical neuropathy on their median nerves and ulnar nerves, respectively, exhibiting confined digit range of motion, reduced grip and pinch strength, decreased tactile sensitivity or slower nerve conduction velocity^5–9^.

Quantitative evaluation of the hand sensorimotor function provides a useful tool for clinical diagnosis of DPN^10^. Accumulating evidence suggests that the effects of DM on hand sensorimotor function could be observed from precision grip – a fundamental grasping manner with opposable thumb and index finger. For example, the patients with DM showed reduced force structural complexity (lower approximate entropy values) than healthy subjects when producing grip force upon a spatially fixed apparatus within 15–50% maximum voluntary contraction (MVC)^11^. Compared to the healthy subjects, the DM patients showed higher force ratio in execution of grip-to-lift task; and the force ratio could serve as indicator to DPN whose sensitivity was 85% and specificity was 51%^10^. The debilitated grip force in DM is associated with reduced tactile sensitivity, blurred afferent inputs and reduced efferent conductivity^10–13^, and is related to the impaired neuromuscular control for hand and digits due to the long-term hyperglycemia^4,14–17^. However, beyond the studies on digit forces, less is known how the hand muscles could be affected by the DM during precision grip.

Multiple muscles are involved and coordinated to generate suitable digit forces for precision grip. Compared with the extrinsic muscles arising outside the hand, the intrinsic muscles that originate and insert within the hand make greater contribution to dexterous manipulation^18,19^. Previous studies have reported that the intrinsic muscles have lower coupling of surface electromyography (sEMG) than extrinsic muscles^18^. Stronger coupling of extrinsic muscles is favorable to synergistic force production, whereas the weaker coupling among intrinsic muscles helps independent control of individual fingers for fine motor tasks^19–21^. It would be an intriguing issue whether the DM impairs the coordination of intrinsic muscles during sustained precision grip that requires continuous sensory inputs and real-time neuromuscular adjustments.

Quantifying intermuscular coordination entails suitable analytical tools. Traditional time-domain approaches, such as the cross-correlation analysis, are based on the magnitude computation and could be easily tampered by abrupt “cross-talks” or additivity noise over the original sEMG waveforms^22,23^. The frequency-domain methods, such as the coherence analysis, work on the cross-spectrum of the sEMG signals and usually show limitations in analyzing the by nature highly complex, nonlinear and nonstationary sEMG signals^24^. Recently, the cross recurrence quantification analysis (CRQA) has been developed as an advanced tool in assessment of dynamical coordination of nonlinear, nonstationary neurophysiological signals^25^. The CRQA provides a set of parameters to quantify the structure of a cross recurrence plot (CRP), which is a visualization of a cross-matrix consisting of all the moments whenever the trajectories of one system pass through the neighborhoods of another system trajectories in the same phase space^25^. Superior to the traditional time- and frequency-domain approaches, the CRQA has advantages in evaluation of the intermuscular coordination as it reveals the dynamical interactions between the two muscles with robustness against nonstationarity transients, model presumption, outliers, and noise^26^. A group of measures derived from CRQA provide quantifications for the deterministic or stochastic components, structural complexity, periodic patterns, or motor synchronization underlying the dynamical coordination across muscles; and these measures can disclose the functionally meaningful features in highly fuzzy, complex, and dynamic control process in neuromuscular systems^25,27^.

This study aimed to investigate the effects of DM on the dynamical coordination of hand intrinsic muscles during precision grip using CRQA. The sEMG signals of the abductor pollicis brevis (APB) and first dorsal interosseous (FDI) were recorded and analyzed using CRQA. In order to examine the neuromuscular control in accordance with impaired sensory inputs with DM, the precision grip was tested by two contrast conditions – the apparatus with stable and with unstable load. The unstable load was supposed to be an effective perturbation for grip control. It was hypothesized that patients with DM would exhibit higher CRQA parameters than the controls during precision grip. It was also hypothesized that the loads could interfere with the intermuscular coordination for patients with DM, and there would be lower CRQA parameters with the unstable load than with the stable load.

## Methods

### Subjects

Thirty-two individuals with Type II DM and the same number of gender- and age-matched healthy subjects participated in the experiment. Subjects’ characteristics are presented in Table 1. All subjects were right-handed with normal or corrected-to-normal vision. The handedness was verified by the Edinburgh Handedness Inventory^28^. The DM patients received clinical diagnosis of Type II DM following the 1997 guideline of American Diabetes Association (ADA). The glycated hemoglobin (HbA1c), fasting plasma glucose (FBG) and post meal blood glucose (PBG) were examined for each DM patients on the day of experiment. The healthy subjects should never be diagnosed or suspected of having hyperglycemia, and their blood sugar levels were tested on spot and should be lower than the criterial level. None of the enrolled subjects reported any history of (1) central nervous system disorders (e.g. multiple sclerosis, Parkinson’s disease, stroke); (2) musculoskeletal or neurological trauma or surgical intervention on their arms and hands; (3) entrapment neuropathies (e.g. cervical spondylosis, brachial plexus injury, shaft tube syndromes or carpal tunnel syndromes); (4) osteoarthritis or rheumatoid arthritis of the hand or wrists. All the subjects were fully informed the purposes of this study and provided written consent prior to the experiment according to the protocols approved by the Institutional Review Board at Shandong University. This study was in accordance with the 1964 Helsinki declaration and its later amendments or comparable ethical standards.

**Table 1:** Characteristics of DM patients and the controls.

Items	Patients	Controls
Gender (Men ~ Women)	23 ~ 9	23 ~ 9
Age (years)	60.2 ± 8.6	60.3 ± 8.8*
Height (cm)	169.4 ± 8.3	167.6 ± 7.0*
Weight (kg)	74.5 ± 12.8	70.9 ± 10.6*
Handedness^(1)^	92.5 ± 15.3	92.3 ± 9.3*
HbA1c (%)^(2)^	8.43 ± 2.41	NA
FBG (mmol/L)^(3)^	10.29 ± 3.34	NA
PBG (mmol/L)^(4)^	13.33 ± 5.43	4.90 ± 1.18
DM Duration (years)	8.8 ± 6.7	0.0 ± 0.0

^(1)^Edinburgh Handedness Inventory; ^(2)^Glycated hemoglobin; ^(3)^Fasting Plasma Glucose; ^(4)^Post meal blood glucose; *No significant difference between the patients and controls (*t*-test, *p* > 0.05).

### Neuromuscular Tests

Potential effects of DM on the neuromuscular system were examined using a group of tests. The neuropathy total symptom score-6 (NTSS-6), the Michigan neuropathy screening instrument (MNSI) and the Michigan hand outcomes questionnaire were three tests to screen and evaluate the symptoms and degrees of diabetic neuropathies, as well as the effect of DM on the hand functions. The fingertip tactile sensitivity of the thumb and index finger was assessed using the Semmes-Weinstein Monofilament tests following a standard protocol^29^. The nerve conduction velocity of the median nerve was assessed for both the sensory and motor pathways. Both the grip and pinch strength values were assessed following a standard testing protocol^30^. All the tests were equally performed on the patients and controls.

### Experimental Set-Up

**Figure 1:**
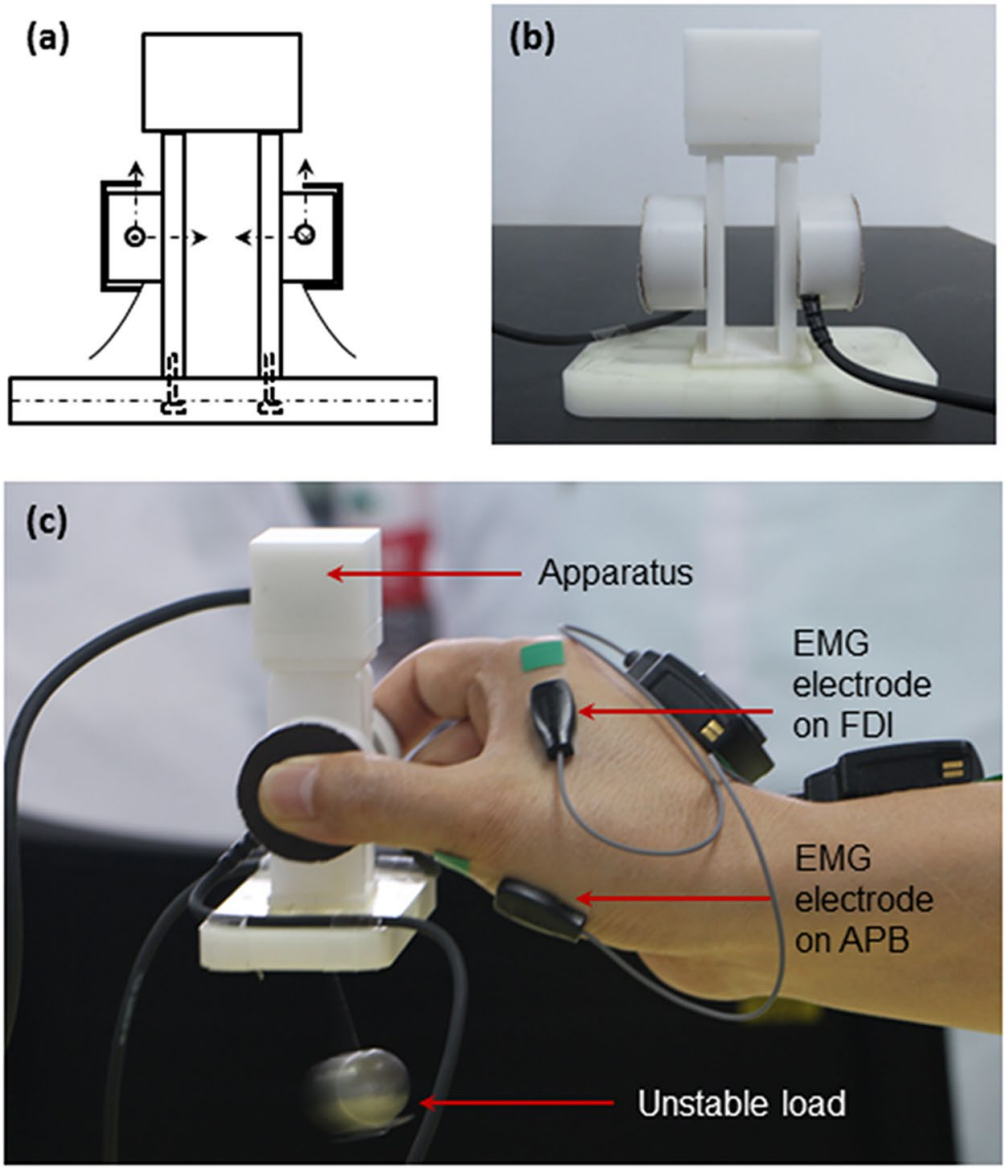
Apparatus, electrodes of sEMG and precision grip with unstable load. (**a**) Close-up of the grip apparatus. (**b**) A photo of the grip apparatus. (**c**) Example grip-trial with unstable load during which the grip force and sEMG signals were recorded simultaneously.

An apparatus was designed to measure the forces of the thumb and index finger during precision grip (Fig. 1). Two miniature 6-component force/torque transducers (Nano17, ATI Industrial Automation, Inc., Apex, NC) were instrumented inside plastic shields for the thumb and index finger, respectively (Fig. 1a,b). The *x*- and *y*-axes were along the vertical and horizontal directions in the surface plane of each transducer, and the *z*-axis was in the perpendicular direction to the contact surface (Fig. 1a). The signals were amplified and multiplexed using custom ATI interface boxes (ATI Industrial Automation, Inc., Apex, NC) and converged to 16-bit analog-digital converters (PCIe-6343, National Instrument, Austin, TX). The signals from the two transducers were recorded, transmitted and processed independently with interactions across channels (Fig. 1b). The pinching surfaces were covered with 100-grit sandpaper and oriented in parallel with a pinch span of 50 mm. A steel ball was rigidly attached below the center of the bottom to offer an extra stable load, or was hung at the center of the bottom with a piece of string to offer an unstable load. The gross weight of the apparatus with the stable/unstable load was 172 g.

Surface EMG signals of the APB and FDI muscles were recorded by two miniature sensors using a wireless EMG system (Trigno^TM^ Mini, Delsys, USA). The EMG system uses silver-contact wireless bipolar bar electrodes with fixed 10 mm inter-electrode spacing. This parallel bar detection approach ensures reliability, robustness to cross-talk, ease-of-use and consistency across all data collection protocols. The mini-electrodes were positioned above the muscle belly parallel to the muscle fibers for the APB and FDI, respectively (Fig. 1c). Positioning of the electrodes was confirmed by an experienced therapist through testing functional movements related to the target muscles following the recommendation^31^. To improve the signal quality, the skin covering the APB and FDI muscles were washed with water and soap, shaved, and cleaned with alcohol. The electrodes were then fixed on the skin with adhesive elastic tape. Proper arrangement of the electrodes could minimize the effects of cross-talk on the target muscles. The sEMG signals were band-pass filtered at 20–450 Hz. Digit force and sEMG collections were implemented using a custom Labview program (National Instrument, Austin, TX). The force and EMG signals were recorded simultaneously at a sampling frequency of 1000 Hz.

### Grip Test Procedures

Each subject sat comfortably in a height adjustable chair at a testing table. The right upper arm was approximately abducted 60° in the frontal plane and flexed 30° in the sagittal plane. The elbow was flexed approximately 120° and the forearm was in a neutral pronation/supination position. The grip test included the following steps. *Session I* – Relaxation. Each subject was required to position their hands on the testing table without any action. Subjects were required to maintain a relaxed state for 1 min, watching their hands during the first 30 s and shutting their eyes for the second 30 s. This session serves as a reference for the following grip sessions. *Session II* – Grip with stable load (SL). Subjects slightly opened their thumb and index finger but closed up the middle, ring and pinky fingers. Once the subject heard a start sign, they reached their grasping hand close to the apparatus, grasped and held it about 30 cm over the table for 1 min. The thumb abducted and internally rotated to establish a posture to oppose the index finger in a dexterous manner^32^. The metacarpophalangeal joint of the thumb was fully extended without flexion observed. The metacarpophalangeal, proximal interphalangeal and distal interphalangeal joints of the index finger flexed about 30°, 45° and 20°. Subjects were instructed to hold the apparatus as stably as they could, maintaining the base of the apparatus horizontally, using a minimum grip force that just prevents the apparatus from slipping. Subjects watched their hands during the first 30 s, and closed their eyes for the other 30 s. After holding it for 1 min, the apparatus was returned back to the initial position. *Session III* – Grip with unstable load (UL). Subjects grasped and held the apparatus with the tips of the thumb and index finger as they did in *Session II* (Fig. 1c). The load (steel ball) with the string in *Session III* formed a pendulum. Given an initial angle, the pendulum oscillated around the center of the base in a simple harmonic motion in sagittal plane (Fig. 1c). There were one trial for both hands in Session I and four trials for each hand in *Session II* and *Session III*. The testing orders for *Session II* and *III*, as well as for the left and right hands, were randomized between subjects. A one-min rest was given between two consecutive trials, and a five-min rest was provided between states. Each subject familiarized with the protocol before the formal tests.

### Data Analysis

**Figure 2:**
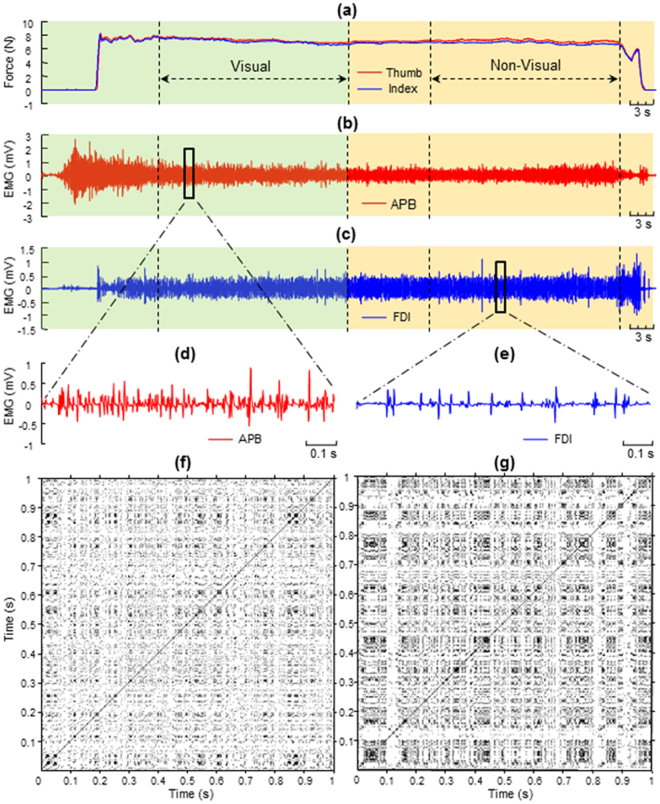
Forces, sEMG signals and recurrence plots from one representative subject. (**a**) Forces of the thumb and index finger in the whole course of precision grip. The phase 20–40 s was the period with visual feedback; and the phase 50–70 s was the period without visual feedback. (**b**,**c**) The sEMG of the APB and FDI at the same time; (**d**,**e**) sEMG of the APB and that of the FDI within 1 s; (**f**,**g**) recurrence plots for the sEMG signals of the APB and FDI within 1 s.

The forces of the thumb and index finger and the sEMG signals from the APB and FDI muscles from one representative subject are depicted in Fig. 2. For both the force and sEMG signals of each trial, the holding phase from 20–40 s with visual feedback and the phase from 50–70 without visual feedback were retained for the following signal processing and statistical analysis (Fig. 2a–c). The mean values and coefficient of variance of the thumb and index finger forces were calculated for the phases with and without visual feedback^33,34^. The root-mean-square (RMS) and median power frequency (MPF) of the EMGs recorded from the APB and FDI were also calculated for the two visual conditions (with vs. without visual feedback).

Recurrence quantification analysis (RQA) was applied to quantify the nonlinear dynamical properties of the APB and FDI contractions. For the *N*-length sEMG series of the APB {*x*(*i*), 1 ≤ *i* ≤ *N*} and that of the FDI {*y*(*i*), 1 ≤ *i* ≤ *N*}, reconstruct the *m*-dimension phase-space vectors $\overrightarrow{u(i)}=\{x(i),x(i+1),...,x(i+m-1)\}\}$ and $\overrightarrow{v(i)}=\{y(i),y(i+1),\mathrm{..}.,y(i+m-1)\}$, respectively, where *i* = 1, …, *N m*. Define the Recurrence Plot (RP) of APB as:

1 $${R}_{i,j}^{m,\varepsilon }={\rm{\Theta }}(\varepsilon \,-\,\Vert \overrightarrow{u(i)}-\overrightarrow{u(j)}\Vert )$$

Similarly, based on formula (1) we can get the RP of FDI by replacing the $\overrightarrow{u(i)}$ by $\overrightarrow{v(i)}$.

The dynamical correlation of the EMG signals recorded from the APB and FDI were analyzed using the CRQA. The CRQA is calculated from the CRP, a graphical representation of a cross matrix defined as:

2 $$C{R}_{i,j}^{m,\varepsilon }={\rm{\Theta }}(\varepsilon -\Vert \overrightarrow{u(i)}-\overrightarrow{v(j)}\Vert )$$

where the *ε* is the predefined threshold, the ${\rm{\Theta }}(\bullet )$ is the Heaviside function and the $\Vert \Vert$ is the Euclidean norm. The following four parameters were derived from the RPs of the APB and FDI, and the CRP: the recurrence rate (RR), the determinism (DET), the entropy of the diagonal lines (ENTR), and the laminarity (LAM)^11^. The RR is defined as

3 $$RR=\frac{1}{{N}^{2}}\sum _{i,j=1}^{N}{R}_{i,j}^{m,\varepsilon }$$

Replace the ${R}_{i,j}^{m,\varepsilon }$ by the $C{R}_{i,j}^{m,\varepsilon }$ defined in (2), then we can get the RR for CRP. The RR indicates the regularity by computing the probability of similar states occurrence in two dynamic systems^25^. Greater RR corresponds to greater correlation in the EMG series. The DET is defined as

4 $$DET=\frac{\sum _{l={l}_{\min }}^{N}l{P}^{\varepsilon }(l)}{\sum _{i,j}^{N}{R}_{i,j}^{m,\varepsilon }}$$

where the ${P}^{\varepsilon }(l)=\{{l}_{i};i=1,\mathrm{..}.,{N}_{l}\}$ is the frequency distribution of the length *l* of diagonal structures and ${N}_{l}$ is the absolute number of diagonal lines with length *l*. The DET is the recurrence points that form diagonal structures to all recurrence points in the RP or CRP, reflecting the deterministic or predictable structures between two dynamic systems^35^. Define the ENTR as:

5 $$\{\begin{array}{c}ENTR=\sum _{l={l}_{{\min }}}^{N}p(l)\,\mathrm{ln}\,p(l)\\ p(l)={P}^{\varepsilon }(l)/\sum _{l={l}_{{\min }}}^{N}{P}^{\varepsilon }(l)\end{array}$$

The ENTR refers to the Shannon entropy of the probability *p(l)* to find a diagonal line having exactly length *l* in RP. It is related to the exponential divergence of the phase space trajectory and correlation entropy, and reflects the complexity of the RP in respect of the diagonal lines^25^. The LAM is defined as:

6 $$LAM=\frac{\sum _{v={v}_{\min }}^{N}v{P}^{\varepsilon }(v)}{\sum _{v=1}^{N}v{P}^{\varepsilon }(v)}$$

The LAM quantifies the laminar phases which is the ratio of recurrence points forming vertical structures to all recurrence points in RP. The distribution ${P}^{\varepsilon }(v)$ of vertical line lengths *v* can be used to quantify laminar phases occurring in a system; and the computation of LAM is realized for those *v* that exceed a minimal length *v*_*min*_. For the recurrence plots of the current study, *v*_*min*_ = 2 is an appropriate value. The denominator summation counts all the recurrence points. Therefore, the LAM will decrease if the RP consists of more single recurrence points while less vertical structures. The LAM demarcates time intervals during which the system’s state is relatively constant compared to intervals of sudden bursts of activity^25,35^.

The parameters of RQA and CRQA, such as embedding dimension or time delay, were determined by both quantitative and empirical ways. First, mutual information (MI) and false nearest neighbors (FNN) were applied to screen the time delay and embedding dimension, respectively. For all trials, the time delay estimated by MI ranged from 1 to 9 but mostly at 1 (64%) and 2 (21%). The embedding dimension estimated by FNN ranged from 1 to 13, with a majority at 1 (82%). To deal with the data with identical parameters, the empirical values were also taken into account^27^. Eventually, the RQA and CRQA was performed on all trials using an embedding dimension of 1, a time delay of 1 sample, and a threshold setting to 10% of the maximum phase space radius. Window with the size 1000 points (1 s) and with an overlap of 200 points (0.2 s) were applied on the signal series to calculate the RQA and CRQA values of each window (Fig. 2d,e). The mean values of all the windows were calculated to indicate the results of the signals. Parameters of RQA and CRQA were implemented with the cross recurrence plot toolbox 5.16 of MATLAB (The Mathworks, Natick, MA, USA). The datasets generated during and/or analyzed during the current study are available from the corresponding author on reasonable request.

### Statistical Analyses

Statistical analyses were performed using SPSS (SPSS Inc., Chicago, IL). Kolmogorov-Smirnov test was applied to examine the data distribution. Independent samples *t*-tests were applied to examine the difference between the DM and controls on neuromuscular functions. To examine differences between DM and controls in digit force performance, analysis of variance (ANOVA) with repeated measures on the mean and coefficient of variation (CV) of digit forces with *Condition* (with versus without visual feedback), *Hand* (the right versus left) and *Digit* (the thumb versus index finger) as within-subject factors and *Group* (DM versus controls) as the between-subject factor, for the states with stable and unstable loads respectively. To quantify the effects of DM on muscle activity, we performed ANOVAs on RMS, MPF, RR, DET, ENTR, LAM with repeated measures with *Condition*, *Hand* and *Muscle* (APB versus FDI) as within-subject factors, and with *Group* as between-subject factor. For the parameters showing significant difference between the DM and controls, a repeated-measures ANOVA with *State* (relax, SL, UL) as within-subject factors and Group as between-subject factor were further applied. The Huynh-Feldt correction was used when the assumption of sphericity was violated. Post-hoc pairwise comparison was performed using the Holm-Sidak test. A *p*-value of less than 0.05 was considered statistically significant.

## Results

Neuromuscular function of the DM and controls are shown in Table 2. The DM group had higher scores in NTSS-6 and MNSI than the controls (*p* < 0.05). The SWM scores of the thumb and index finger of both the left and right hands of DM were significantly higher than those of the controls (*p* < 0.05). The DM group showed a reduction in the NCV along the left and right median nerves (motor: *p* < 0.001; sensory: *p* < 0.05). No significant difference was observed between the two groups in MHQ (left: *p* = 0.176; right: *p* = 0.279), grip strength (left: *p* = 0.138; right: *p* = 0.187) or pinch strength (left: *p* = 0.310; right: *p* = 0.580).

**Table 2:** Neuromuscular tests scores of the DM patients and the controls.

Neuromuscular Tests			Patients	Controls
NTSS-6^(1)^			1.64 ± 2.40	0.40 ± 0.53*
MNSI^(2)^			1.38 ± 1.24	0.72 ± 0.77*
MHQ^(3)^		Left	98.54 ± 2.54	99.28 ± 1.67
		Right	98.86 ± 2.46	99.43 ± 1.61
SWM^(4)^	Thumb	Left	3.58 ± 0.36	3.28 ± 0.58*
		Right	3.62 ± 0.40	3.34 ± 0.52*
	Finger	Left	3.48 ± 0.42	3.06 ± 0.49**
		Right	3.48 ± 0.42	3.16 ± 0.57*
NCV^(5)^	Motor	Left	51.56 ± 5.16	57.62 ± 3.79**
		Right	51.78 ± 4.38	56.09 ± 4.25**
	Sensory	Left	56.00 ± 13.18	62.53 ± 7.75*
		Right	56.13 ± 16.06	62.94 ± 6.28*
Grip Strength (kg)		Left	31.60 ± 8.04	34.64 ± 8.20
		Right	33.89 ± 9.08	36.93 ± 9.19
Pinch Strength (kg)		Left	5.19 ± 1.23	5.52 ± 1.38
		Right	5.44 ± 1.69	5.69 ± 1.78

^(1)^Neuropathy total symptom score-6; ^(2)^Michigan neuropathy screening instrument; ^(3)^Michigan hand outcomes questionnaire; ^(4)^Semmes-Weinstein Monofilament; ^(5)^Nerve conduction velocity; *Significant difference between the patients and controls (*t*-test, *p* < 0.05); **Significant difference between the patients and controls (*t*-test, *p* < 0.001).

The mean and CV of the thumb and index finger forces with stable and unstable loads are shown in Table 3. The repeated measures ANOVA did not show any significant difference between the DM and controls for either the mean (SL: *p* = 0.355; UL: *p* = 0.415) or CV (SL: *p* = 0.431; UL: *p* = 0.132). The within-subject factors, such as the hands, visual conditions, showed significant effects on the mean and CV of digit forces. The mean force had significant difference between the right and left hands (*F*_1,62_ = 4.114, *p* < 0.05), between the visual and non-visual conditions (*F*_1,62_ = 45.450, *p* < 0.001), and between the thumb and index finger (*F*_1,62_ = 11.828, *p* < 0.05) with SL; and between the visual and non-visual conditions (*F*_1,62_ = 55.915, *p* < 0.001) and between the two digits (*F*_1,62_ = 20.450, *p* < 0.001) with UL. The CV showed significant differences between hands (*F*_1,62_ = 5.974, *p* < 0.05) and between visual conditions (*F*_1,62_ = 49.113, *p* < 0.001) with SL; and between the visual and non-visual conditions (*F*_1,62_ = 56.912, *p* < 0.001) with UL.

**Table 3:** Forces of the thumb and index finger during precision grip. SL: Grip with stable load; UL: Grip with unstable load. Data were presented as mean ± standard deviation.

			Visual				Non-Visual			
			Left		Right		Left		Right	
			Thumb	Finger	Thumb	Finger	Thumb	Finger	Thumb	Finger
SL	Mean	Patient	6.16 ± 1.99	6.18 ± 1.98	6.60 ± 2.17	6.50 ± 2.15	5.81 ± 1.93	5.83 ± 1.92	6.24 ± 1.97	6.12 ± 1.94
		Control	5.75 ± 2.20	5.78 ± 2.16	6.17 ± 2.15	6.11 ± 2.11	5.32 ± 1.97	5.36 ± 1.93	5.77 ± 1.94	5.70 ± 1.90
	CV	Patient	4.38 ± 2.18	4.36 ± 2.20	3.78 ± 1.41	3.81 ± 1.38	3.45 ± 1.67	3.42 ± 1.70	2.96 ± 1.42	2.95 ± 1.35
		Control	4.24 ± 1.85	4.18 ± 1.84	3.74 ± 1.65	3.77 ± 1.61	2.94 ± 1.52	2.85 ± 1.42	2.72 ± 1.40	2.68 ± 1.36
DL	Mean	Patient	7.27 ± 2.11	7.27 ± 2.11	7.43 ± 2.53	7.32 ± 2.51	6.55 ± 1.97	6.55 ± 1.96	7.80 ± 2.19	6.69 ± 2.17
		Control	6.71 ± 2.51	6.74 ± 2.48	6.93 ± 2.47	6.82 ± 2.41	6.20 ± 2.19	6.22 ± 2.17	6.71 ± 2.29	6.31 ± 2.22
	CV	Patient	6.16 ± 3.73	6.18 ± 3.78	5.94 ± 3.13	6.04 ± 3.17	4.39 ± 2.63	4.35 ± 2.64	4.18 ± 2.23	4.18 ± 2.18
		Control	5.37 ± 2.55	5.36 ± 2.55	4.76 ± 2.20	4.81 ± 2.14	3.69 ± 1.60	3.64 ± 1.59	3.86 ± 1.93	3.86 ± 1.90

The repeated measures ANOVA showed that the DM did not affect the RMS (SL: *p* = 0.794; UL: *p* = 0.932) or the MPF (SL: *p* = 0.829; UL: *p* = 0.778) of sEMG (Table 4). Significant differences of RMS were observed between the visual and non-visual conditions (SL: *F*_1,62_ = 33.146, *p* < 0.001; UL: *F*_1,62_ = 55.026, *p* < 0.001) and between the APB and FDI (SL: *F*_1,62_ = 38.667, *p* < 0.001; UL: *F*_1,62_ = 28.940, *p* < 0.001). Similarly, there was significant difference in MPFs between the visual and non-visual conditions (SL: *F*_1,62_ = 19.569, *p* < 0.001; UL: *F*_1,62_ = 45.033, *p* < 0.001) and between the APB and FDI (SL: *F*_1,62_ = 35.434, *p* < 0.001; UL: *F*_1,62_ = 21.755, *p* < 0.001). No significant difference was found between the left and right hand for either the RMS (SL: *p* = 0.887; UL: *p* = 0.923) or MPF (SL: *p* = 0.059; UL: *p* = 0.065).

**Table 4:** The sEMG RMS and MPF of the APB and FDI.

			Visual				Non-Visual			
			Left		Right		Left		Right	
			APB	FDI	APB	FDI	APB	FDI	APB	FDI
SL	RMS( × 0.1	Patient	0.94 ± 0.53	0.53 ± 0.36	0.92 ± 0.76	0.55 ± 0.41	0.77 ± 0.42	0.56 ± 0.37	0.75 ± 0.56	0.55 ± 0.38
		Control	1.03 ± 0.65	0.43 ± 0.35	1.10 ± 0.74	0.37 ± 0.27	0.87 ± 0.55	0.36 ± 0.28	0.85 ± 0.55	0.35 ± 0.23
	MPF	Patient	158.04 ± 22.81	153.16 ± 28.52	150.24 ± 26.58	145.52 ± 29.98	143.51 ± 19.00	141.99 ± 16.45	137.49 ± 18.78	138.34 ± 18.27
		Control	153.07 ± 22.70	147.68 ± 25.11	155.33 ± 26.67	151.16 ± 25.58	145.16 ± 19.23	141.51 ± 19.68	135.05 ± 27.67	132.07 ± 26.75
DL	RMS( × 0.1)	Patient	0.85 ± 0.47	0.59 ± 0.45	0.89 ± 0.69	0.50 ± 0.037	0.71 ± 0.38	0.55 ± 0.42	0.73 ± 0.58	0.48 ± 0.36
		Control	0.96 ± 0.53	0.46 ± 0.23	1.11 ± 0.73	0.39 ± 0.025	0.75 ± 0.38	0.44 ± 0.24	0.86 ± 0.60	0.38 ± 0.28
	MPF	Patient	157.97 ± 22.96	154.80 ± 23.00	150.21 ± 30.61	144.16 ± 32.44	147.22 ± 19.25	143.04 ± 18.69	140.04 ± 17.93	136.42 ± 20.71
		Control	158.59 ± 21.12	149.46 ± 26.19	157.79 ± 29.96	150.94 ± 30.01	145.37 ± 16.81	140.69 ± 20.99	141.99 ± 23.34	138.56 ± 22.22

SL: Grip with stable load; UL: Grip with unstable load. Data were presented as mean ± standard deviation.

**Figure 3:**
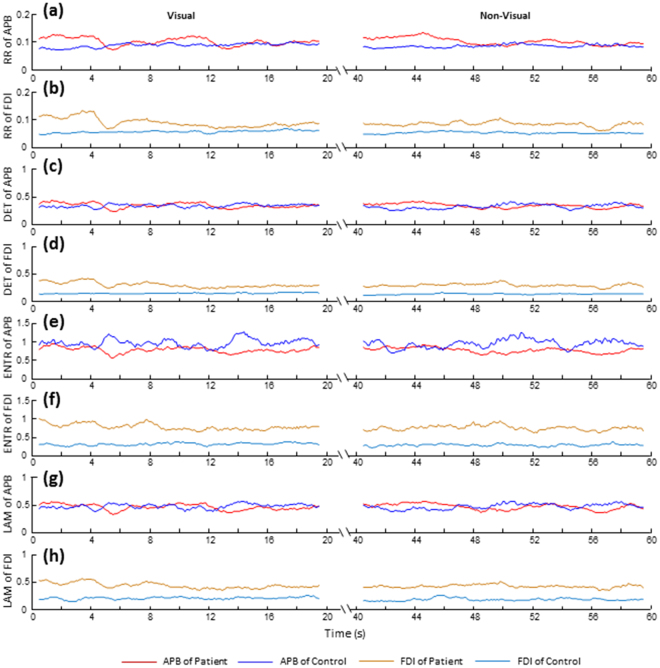
Time series of the RQA parameters calculated from the APB and FDI muscles during precision grip from a representative DM patient and the control. (**a,c,e,g**) RR, DET, ENTR and LAM of the APB; (**b,d,f,h**) RR, DET, ENTR and LAM of the FDI. A stable loads was provided for this trial.

**Figure 4:**
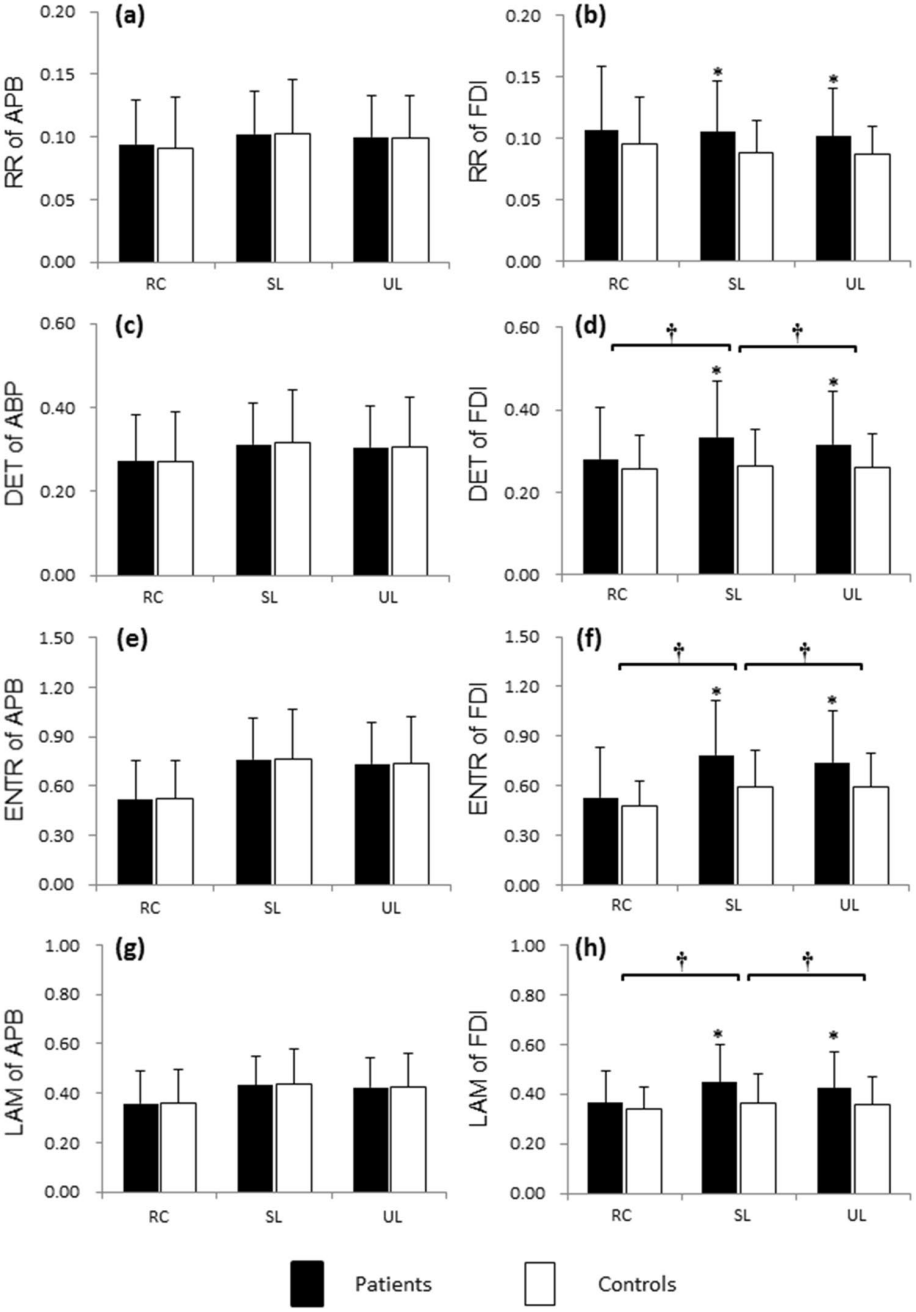
Statistical analysis of RQA parameters of the APB and FDI for precision grip. (**a,c,e,g**) RR, DET, ENTR and LAM of the APB under the relaxed, stable load and unstable load conditions; (**b,d,f,h**) RR, DET, ENTR and LAM of the FDI under the relaxed condition (RC), with stable load (SL) and unstable load (UL). *Significant difference between the patients and controls (*p* < 0.05). ^†^Significant difference across the load conditions (*p* < 0.05).

The RPs corresponding to the APB and FDI muscle contractions within 1 s (shown in Fig. 2d,e) are depicted in Fig. 2f and g, respectively. The RQA parameters with time from one representative DM patient and the control subject are illustrated in Fig. 3. Statistical results of RQA parameters of APB and FDI in DM patients and controls during precision grip without visual feedback are shown in Fig. 4. The DM had higher RR (SL: *F*_1,62_ = 5.721, *p* < 0.05; UL: *F*_1,62_ = 4.572, *p* < 0.05, Fig. 4b), DET (SL: *F*_1,62_ = 8.585, *p* < 0.01; UL: *F*_1,62_ = 6.257, *p* < 0.05, Fig. 4d), ENTR (SL: *F*_1,62_ = 10.694, *p* < 0.01; UL: *F*_1,62_ = 7.725, *p* < 0.01, Fig. 4f), and LAM (SL: *F*_1,62_ = 8.838, *p* < 0.01; UL: *F*_1,62_ = 5.784, *p* < 0.05, Fig. 4h) of FDI than the controls. By contrast, no significant difference was found between the DM and controls in RQA parameters of APB with SL (RR: *p* = 0.963; DET: *p* = 0.812; ENTR: *p* = 0.934; LAM: *p* = 0.957) or UL (RR: *p* = 0.992; DET: *p* = 0.914; ENTR: *p* = 0.984; LAM: *p* = 0.960, Fig. 4a,c,e,g). Compared to the relaxed condition, holding the apparatus with either SL or UL led to a significant increase in the DET, ENTR and LAM of both the APB and FDI (*p* < 0.05, Fig. 4). For the patients with DM, precision grip with UL showed significantly lower DET, ENTR and LAM of the FDI than with SL (*p* < 0.05, Fig. 4). No significant difference between the SL and UL conditions in the RQA parameters of APB (*p* > 0.05, Fig. 4).

**Figure 5:**
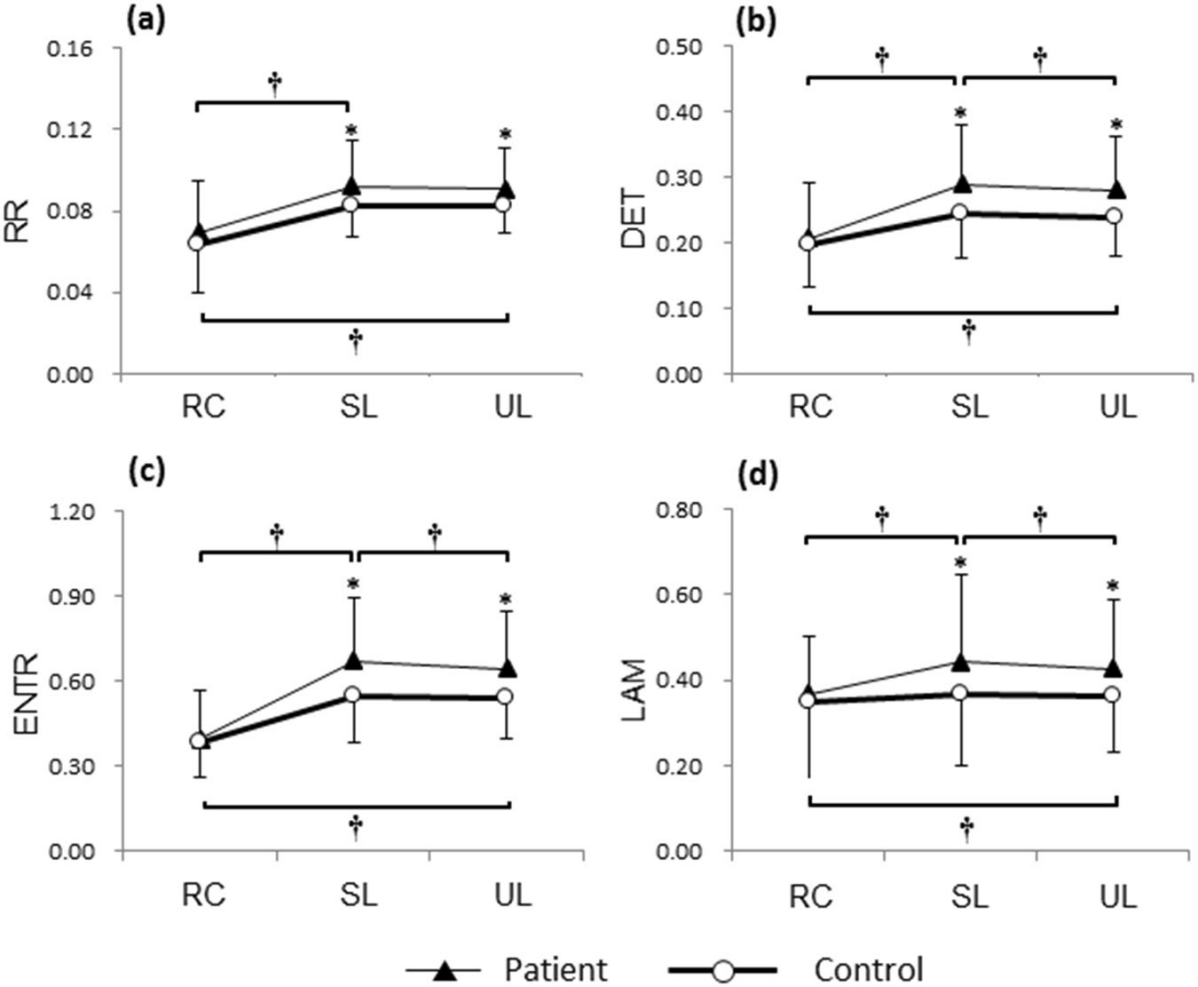
Statistical analysis of CRQA parameters across the APB and FDI. (**a,b,c,d**) RR, DET, ENTR and LAM of the patients with DM and the controls under the relaxed condition (RC), with stable load (SL) and unstable load (UL). *Significant difference between the patients and controls (*p* < 0.05). ^†^Significant difference across the load conditions (*p* < 0.05).

Statistical results of CRQA parameters across the APB and FDI during precision grip are shown in Fig. 5. The DM showed higher RR (SL: *F*_1,62_ = 5.355, *p* < 0.05; UL: *F*_1,62_ = 5.465, *p* < 0.05), DET (SL: *F*_1,62_ = 6.691, *p* < 0.05; UL: *F*_1,62_ = 7.304, *p* < 0.01), ENTR (SL: *F*_1,62_ = 8.201, *p* < 0.01; UL: *F*_1,62_ = 8.320, *p* < 0.01), and LAM (SL: *F*_1,62_ = 8.505, *p* < 0.01; UL: *F*_1,62_ = 6.990, *p* < 0.01) than the controls. Holding the apparatus with SL or UL the subjects had higher DET, ENTR and LAM across the sEMGs of APB and FDI than being relaxed (*p* < 0.05, Fig. 5). For the patients with DM, grasping with UL led to lower DET, ENTR and LAM across the sEMGs of APB and FDI than with SL (*p* < 0.05, Fig. 5). No significant difference between the SL and UL conditions was found in the CRQA parameters of the controls.

## Discussion

This study examined the effects of DM on the dynamical coordination of intrinsic muscles during precision grip. Compared to the healthy subject, the patients with DM had higher blood sugar level (e.g. the HbA1c and PBG in Table 1), reduced digit-tip tactile sensitivity (e.g. the SWM in Table 2), lower motor and sensory nerve conduction velocity along the median nerve (Table 2), more neuropathy symptoms (e.g. the NTSS-6 and MNS in Table 2) and impaired overall hand function (MHQ in Table 2). The left and right hand showed comparable reduction in tactile sensation and sensory and motor conductivity via the median nerve, which confirmed the bilateral development of neuropathy – a typical manifestation in DPN^36,37^. No significant difference was observed in grip or pinch strength between the DM patients and the controls (Table 2). Although several studies have reported that the DM performed relatively lower grip force than controls^16,38–40^, Gorniak *et al*. argued that the subject characteristics in most of the previous studies were loosely controlled and led to contradictory conclusions about the effects of DM on motor ability (e.g. the grip and pinch strength)^13^. In their study, the DM patients and the controls did not show significant difference in their pinch forces^13^. More studies support the notion that the DPN at early stage could be associated with deficits of sensory afferents but not necessarily with detectable reduction in grip strength^41,42^.

Result showed that the DM did not affect the amount (mean) or variability (CV) of the thumb and index finger forces during stable precision grip (Table 3). Considering the precision grip is a sensorimotor process, the motor systems are under both feedforward and feedback controls. The feedforward mechanism allows individuals to program appropriate motor commands prior to grasping according to previous experiences of the object properties; whereas the feedback mechanism adjusts neuromuscular activations according to real-time sensory information^27,34,43^. By examining the grip force performance in process of holding to lifting an object, Chiu *et al*. found that the DM patients exhibited reduction in capacity of online digit-force adjustment according to the inertial load during the object’s dynamic movement^10^. The current study revealed that holding the object stably in air might exempt the digit force exertion from online feedback control based on peripheral sensory inputs, but rather rely on a feedforward control based on pre-programmed motor commands or default modes for force production^44^. It could be inferred that the feedforward control could extensively compensate for the loss of sensory information associated with the DM, guaranteeing the patients and controls with similar digit force production for the stable precision grip task.

Results showed that when grasping and holding a light object (the gross weight of apparatus was only 170 g), the patients with DM and the controls maintained roughly the same digit force levels and variability, as well as the similar muscle contraction magnitudes (RMS) and frequency (MPF, Table 4). Interestingly, based on the comparable force outputs and similar levels of muscle activation, patients with DM and the controls exhibited quite different dynamical patterns for intermuscular coordination (Figs 3–5). The DM patients had significantly higher CRQA parameters (RR, DET, ENTR and LAM) than the controls (Fig. 5). The increased RR shows a higher probability of similar states occurrence in the neuromuscular dynamic systems, revealing a higher regularity in the intermuscular dynamical coupling. The increased DET indicates a more deterministic structure (or predictable structures) of intermuscular coordination in the DM patients compared to the controls. The increased ENTR in DM reflects augmented complexity of the deterministic structure during coupling of the muscles. The higher LAM represents the increased occurrence of laminar states in the neuromuscular systems of the APB and FDI, implying more constant firing rate and reduced instability in the neuromuscular control in DM. In addition, the RQA results from individual muscles showed that the DM patients had significantly higher RR, DET, ENTR and LAM than the controls from the FDI’s sEMG signals (Fig. 4b,d,f,h); by contrast, no significant difference between the patients and controls were found in the RQA indicators of the APB’s sEMG signals (Fig. 4a,c,e,g). This finding suggests that the altered intermuscular coordination associated with DM would be highly related to the changes in neuromuscular activation of FDI. Previous electrophysiological studies have found that the patients with DPN had about 30% reduction of motor unit number estimates, 20% reduction of compound muscle action potentials and 15% reduction of mean firing rates on their FDI muscle compared to the healthy subjects, leading to muscular remodeling and altered firing patterns^45^. The degeneration and dysfunction of FDI associated with DPN would present in the dexterous hand task and significantly affect the dynamical structure of muscle activity during sustained precision grip. It should be noted that the effects of DM reflected by RQA (Fig. 4) and CRQA (Fig. 5) were associated with muscle contractions; otherwise, at relaxed state without muscle contraction, no significant difference was observable between the DM and control groups. This may further confirm the neurophysiological significance of the information captured by RQA and CRQA, particularly in reflection of the DM-related influences.

The CRQA is an analytical tool to identify functionally meaningful actions in fuzzy, complex, and dynamic neuromuscular activities, such as deterministic or stochastic components, structural complexity, periodic patterns, or motor synchronization, underlying the dynamical coordination across the APB and FDI muscles that contribute to prehensile kinetics^25^. The anatomical and neural arrangement of the APB and FDI muscles substantiates the two as relatively interdependent systems that strongly couple and intelligently match each other during precision grip^46,47^. This study found that the DM patients had significantly higher CRQA parameters (RR, DET, ENTR and LAM) than the controls (Fig. 5), suggesting altered dynamical coordination across muscles. A compensatory mechanism underlying grip control may explain for this phenomenon^27,33^. With long-term high blood glucose, sensory feedback of DM patients was intensely obstructed (Table 2) so that subjects need to compensate for the loss of sensory feedback by relying more on the feedforward control. Under this compensatory mechanism, preprogrammed motor commands were reinforced but the online feedback regulation was diminished, rendering more deterministic structures for the APB-FDI coordination.

This study examined precision grip with UL in addition to SL. The purpose to set the UL condition is to provide a perturbation by which the effects of DM on precision grip control would be magnified. Results showed that precision grip with UL had significantly higher DET, ENTR and LAM for the FDI (Fig. 4d,e,f), and higher CRQA parameters (DET, ENTR and LAM) across the APB and FDI (Fig. 5b,c,d), in comparison of the grip with SL. This revealed that the load perturbation could remarkably interfere with the FDI contraction and intermuscular coordination between the two intrinsic muscles during sustained precision grip. This finding is in line with the previous studies that both the intrinsic muscle activation and muscle synergy for precision grip are under modulation of environmental factors^48–50^. It is noteworthy that the differences in RQA and CRQA parameters between the UL and SL were observed in patients with DM rather than in the controls, suggesting that the patients with DM had lower adaptability to the uncertainty of environment than the healthy individuals. Specifically, as grasping and interacting with the object with UL, the digits need to produce suitable forces to the changing center of gravity of the apparatus, thereby demanding higher level of online regulation than grasping an object with SL^51,52^. The higher RQA and CRQA values in DM with UL indicate a more regular organization of motor potentials in FDI and a stronger intermuscular coupling between APB and FDI than with SL. These findings provide evidence that the sensory deficits in DM could remarkably decrease the capacity of online motor adjustment based on sensory feedback, making the grip control rely more on the feedforward strategy.

The APB and FDI were selectively examined in this study since they are key intrinsic hand muscles involved in precision grip tasks and are reflective of abundant neurophysiological information^53,54^. Relative to the other intrinsic muscles the ABP and FDI muscles are easy to access by the surface EMG electrodes. In literature, the APB and FDI are a muscle pair that has been frequently examined, particularly in the studies focusing on the intermuscular coherence or neural drive coordination for dexterous manual tasks^46,55^. It is noteworthy that more muscles, including the intrinsic muscles like the flexor pollicis brevis and opponens pollicis and the extrinsic muscles like the abductor policis longus and extensor pollicis brevis, may also contract synergistically with the APB during precision grip. Activation of the synergistic muscles may share the motor commands delivering to the target muscles and alter the regulation of motor unit recruitment for the specific muscle. This could partially explain the results that no significant effects of DM was found on the APB activation (Fig. 4a,c,d,g). Previous studies found there are probably no unique and deterministic synergistic muscle activation pattern in low force range during precision grip^47^. It would be essential to examine more synergistic muscles to better understand how they interact with the APB and how the synergistic contraction is affected by DM with higher level of force production.

This study investigated the effects of DM on dynamical muscle coordination underlying sensorimotor control for a functionally meaningful action. A freely moveable apparatus was chosen instead of a spatially fixed handle. By this set-up the thumb and index finger need to comply with mechanical or task constraints such as producing zero residual forces and moments; guaranteeing enough load force to counterbalance the weight of the handle; avoiding handle tilt from unbalanced forces across the digits, and accommodate to the load perturbations, etc. To fulfill all these constraints the motor system needs to produce well-coordinated digit forces^34,56^. The DM-related patterns observed in the dynamical muscle coordination were associated with the inter-digit force coordination during precision grip of a freely moveable apparatus. It would be of interest to further examine the effects of DM on the dynamical muscle coordination when individuals pinch upon a spatially fixed apparatus with less coupled digit forces. This may help us learn more about the associations between each muscle activation and the specific digit force production.

## Conclusions

The DM changed the dynamical structures of muscle activation for the FDI and of the intermuscular coordination between the APB and FDI during stable precision grip. More deterministic structures were found in the sEMG signals of FDI in DM patients, potentially attributable to the muscular remodeling and altered firing patterns associated with DM. A compensatory feedforward mechanism may be responsible for the stronger intermuscular coupling between the APB and FDI muscles. Sensory deficits in DM remarkably decreased the capacity of online motor adjustment based on sensory feedback, rendering a decreased adaptability to the uncertainty of environment. This study shed light on the inherent dynamical properties underlying the intrinsic muscle activation and intermuscular coordination, and the role of DM on sensorimotor function. Findings of this study may facilitate the development of a non-invasive method for clinical diagnosis of DPN.
